# Supplementary material for: RNA supply drives physiological granule assembly in neurons
Source: Nat Commun. 2022 May 19;13:2781. doi: 10.1038/s41467-022-30067-3 (PMC9120520; doi:10.1038/s41467-022-30067-3)

Supplementary information

RNA supply drives physiological granule assembly in neurons

Karl E Bauer[1], Niklas Bargenda[1], Rico Schieweck[1], Christin Illig[1], Inmaculada Segura[1,2], Max Harner[1], and Michael A Kiebler[1]

[1] BioMedical Center, Dept. Cell Biology and Anatomy, Medical Faculty, Ludwig Maximilians University, Großhaderner Str. 9, 82152 Planegg-Martinsried, Germany

[2] Current address: Max Planck Institute for Biological Intelligence (in foundation), Am Klopferspitz 18, 82152 Martinsried, Germany

This file contains:

- 5 Supplementary Figures
- 5 Supplementary Figures legends
- 3 Supplementary Movie legends

  (movies provided separately on journal website)

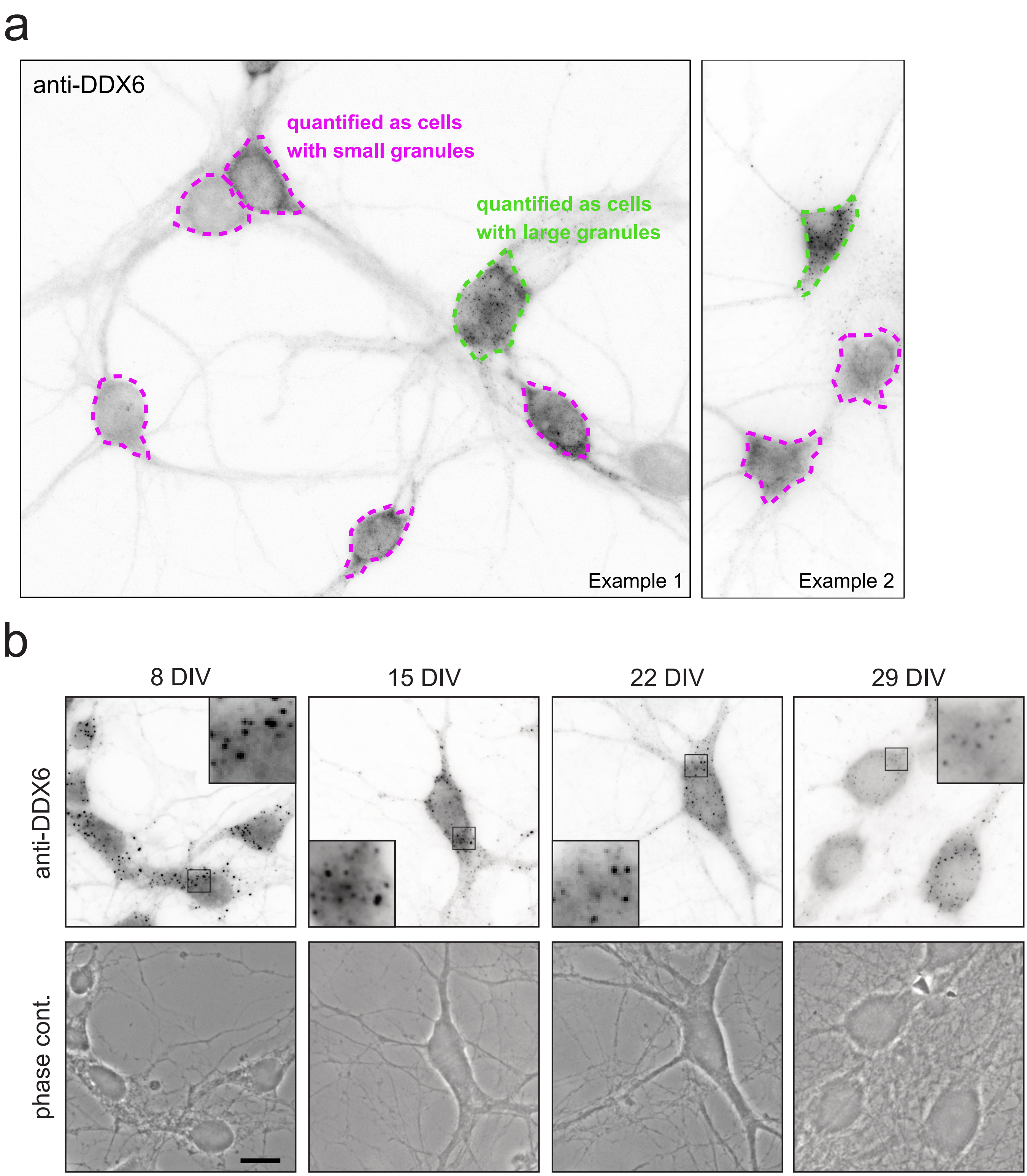

Supplementary Figure 1 - Bauer et al.

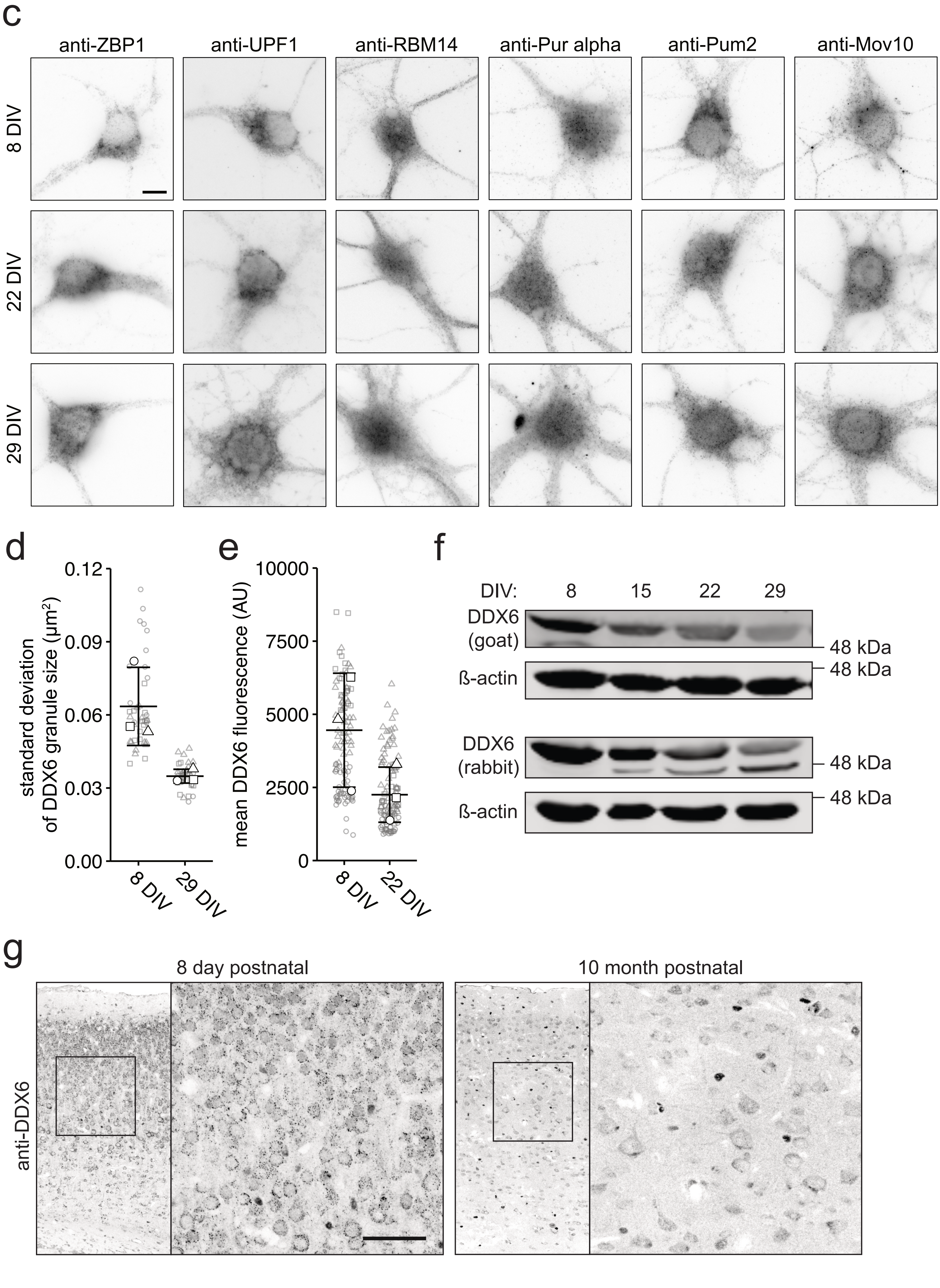

Supplementary Figure 1 (continued) - Bauer et al.

h

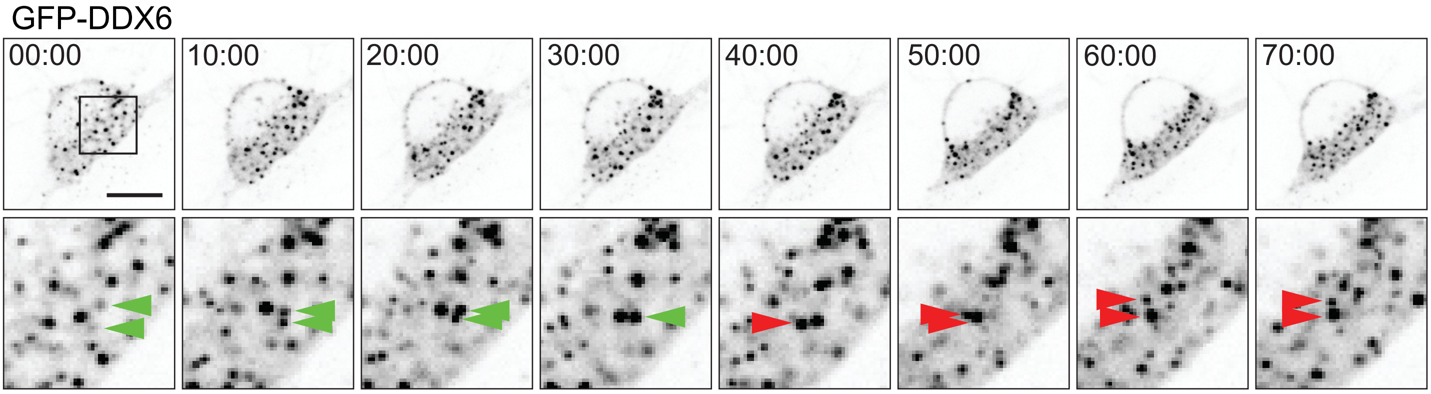

(see also movie 1)

Supplementary Figure 1: Cytoplasmic DDX6 granule disassembly during neuronal maturation occurs in hippocampal and cortical neurons and is accompanied by a reduction of DDX6 protein levels. (**a**) Representative examples of manual quantification of cell population in DDX6 immunostainings. Representative cells classified as containing small granules are outlined in magenta, representative cells classified as containing large granules are outlined in green. (**b**) Representative examples of DDX6 immunostainings and phase contrast pictures of 8, 15, 22 and 29 DIV cortical neurons in culture. Boxed regions in images are displayed as magnified insets. (**c**) Representative examples of ZBP1, UPF1, RBM14, Pur alpha, Pum2 and Mov10 immunostainings of 8, 22, and 29 DIV hippocampal neurons. (**d, e**) Dot plots displaying the standard deviation of DDX6 granule size (d) and mean DDX6 fluorescence of individual cell bodies (e) in 8, 29 or 22 DIV hippocampal neurons in culture. Small grey symbols represent single cells while larger white symbols indicate the average of each replicate. Distinct dot symbols indicate biological replicates. Horizontal line and error bars represent mean of replicates and standard deviation (n=3 biologically independent experiments). AU = arbitrary units. (**f**) Representative Western blots of cortical neuron lysates at 8, 15, 22 and 29 DIV, respectively, decorated with goat-anti-DDX6 (upper) or rabbit-anti-DDX6 (lower) and anti-ß-actin antibodies as loading control. (**g**) DDX6 immunostainings of sagittal brain tissue slices in the cortex of 8 day and 10 month postnatal mice. Boxed regions in overviews show location of magnified region. This experiment was repeated independently 3 times with similar results. (**h**) Representative time series of a 11 DIV hippocampal neuron transfected with GFP-DDX6. Green arrowheads indicate fusing granules, red arrowheads indicate splitting granules. Timestamp indicates minutes:seconds. Boxed regions indicate area of magnified insets displayed below. See also Suppl. Movie 1. Scale bars 10 μm in (b), (c) and (g), 50 μm in (g). *Related to Figure 1*.

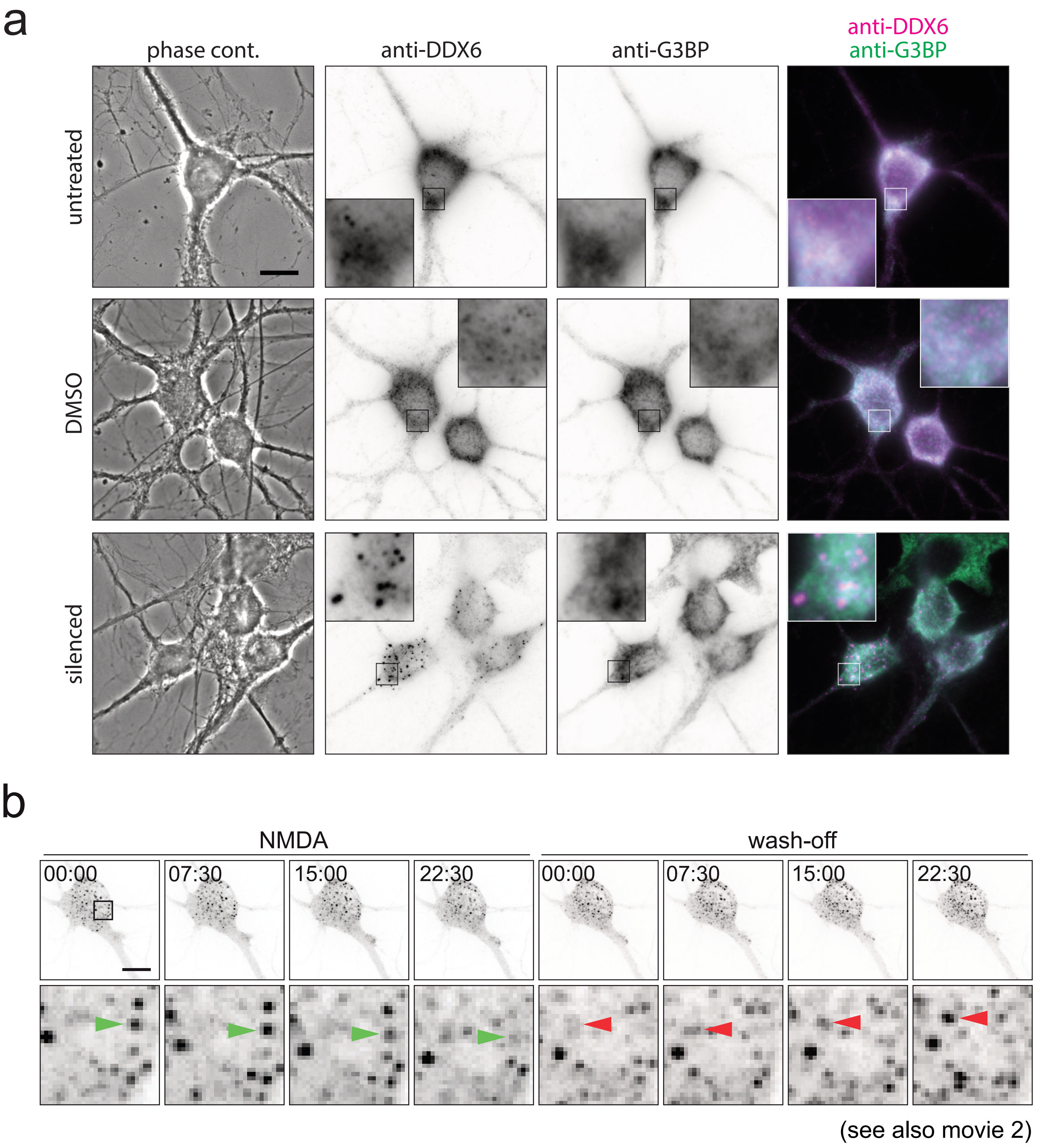

SUPPLEMENTARY FIGURE 2 - Bauer et at.

Supplementary Figure 2: DDX6 granules induced by chemical inhibition of neuronal activity are not stress granules. (**a**) Representative examples of DDX6, G3BP immunostainings and phase contrast pictures of 22 DIV hippocampal neurons in culture under untreated, vehicle treated or silenced (100μM CNQX, 50μM AP5, 1μM TTX) conditions. Boxed regions in images are displayed as magnified insets. (**b**) Representative time series of a 16 DIV hippocampal neuron transfected with GFP-DDX6 during incubation with 100μM NMDA or subsequent wash-off. Green arrowheads indicate disassembly of an individual granule, red arrowheads indicate assembly of an individual granule. Timestamp indicates minutes:seconds. Boxed regions indicate area of magnified insets displayed below. Scale bars 10 μm. See also Suppl. Movie 2. *Related to Figure 2*.

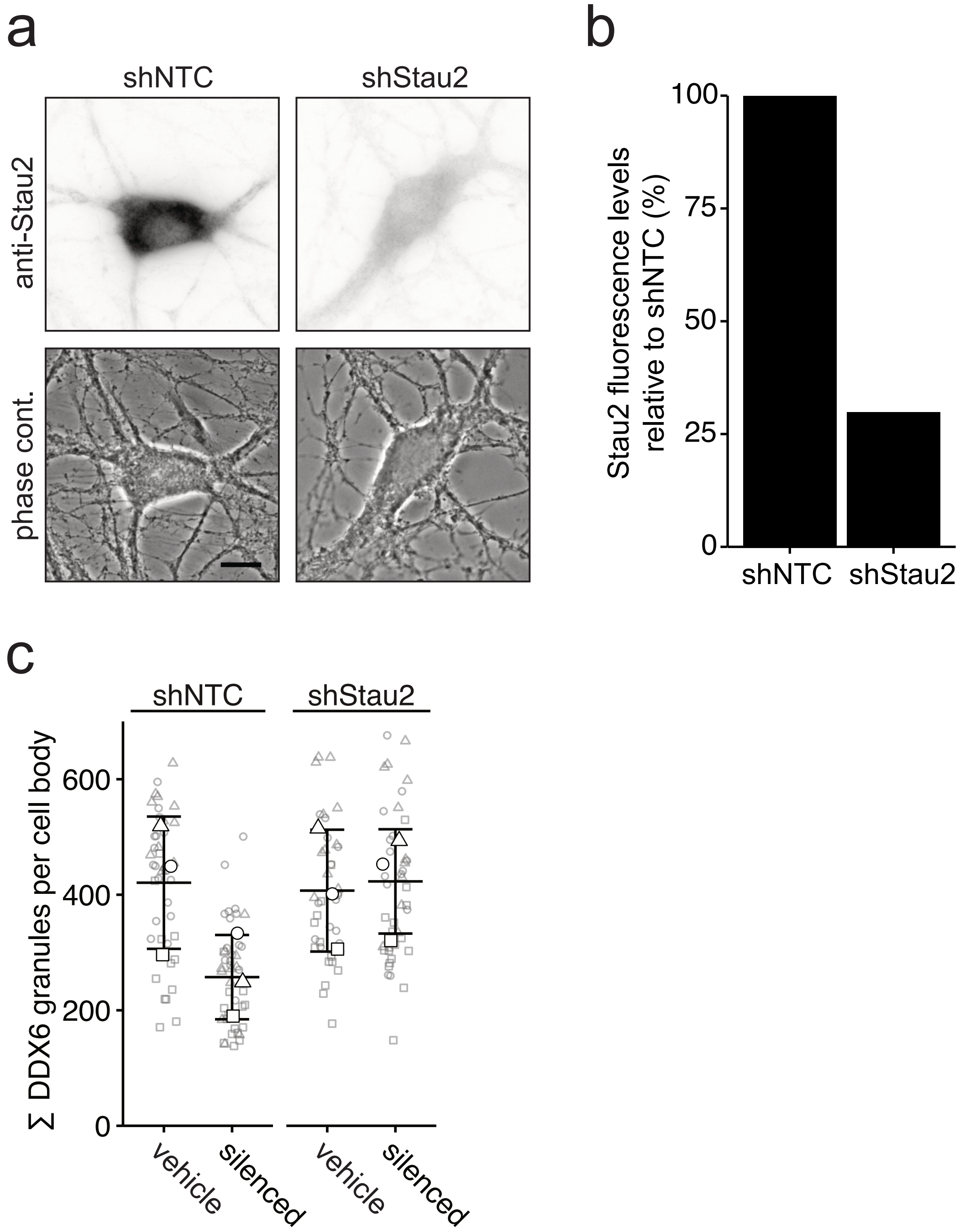

Supplementary Figure 3 - Bauer et al.

Supplementary Figure 3: Transduction of an shStau2 expressing lentivirus results in efficient downregulation of Stau2. (**a**) Representative 22 DIV hippocampal neurons 4 days after transduction with either shNTC or shStau2 lentiviral particles stained with anti-Stau2 antibodies. (**b**) Bar plot displaying relative Stau2 intensity normalized to shNTC in cell bodies of 22 DIV rat hippocampal neurons 5 days after viral transduction with either shNTC or shStau2 lentiviral particles. (**c**) Dot plot displaying DDX6 granule number of individual cell bodies. Distinct dot symbols indicate biological replicates. Small grey symbols represent single cells while larger white symbols indicate the average of each replicate. Horizontal line and error bars represent mean of replicates and standard deviation (n=3 biologically independent experiments). *Related to Figure 3*.

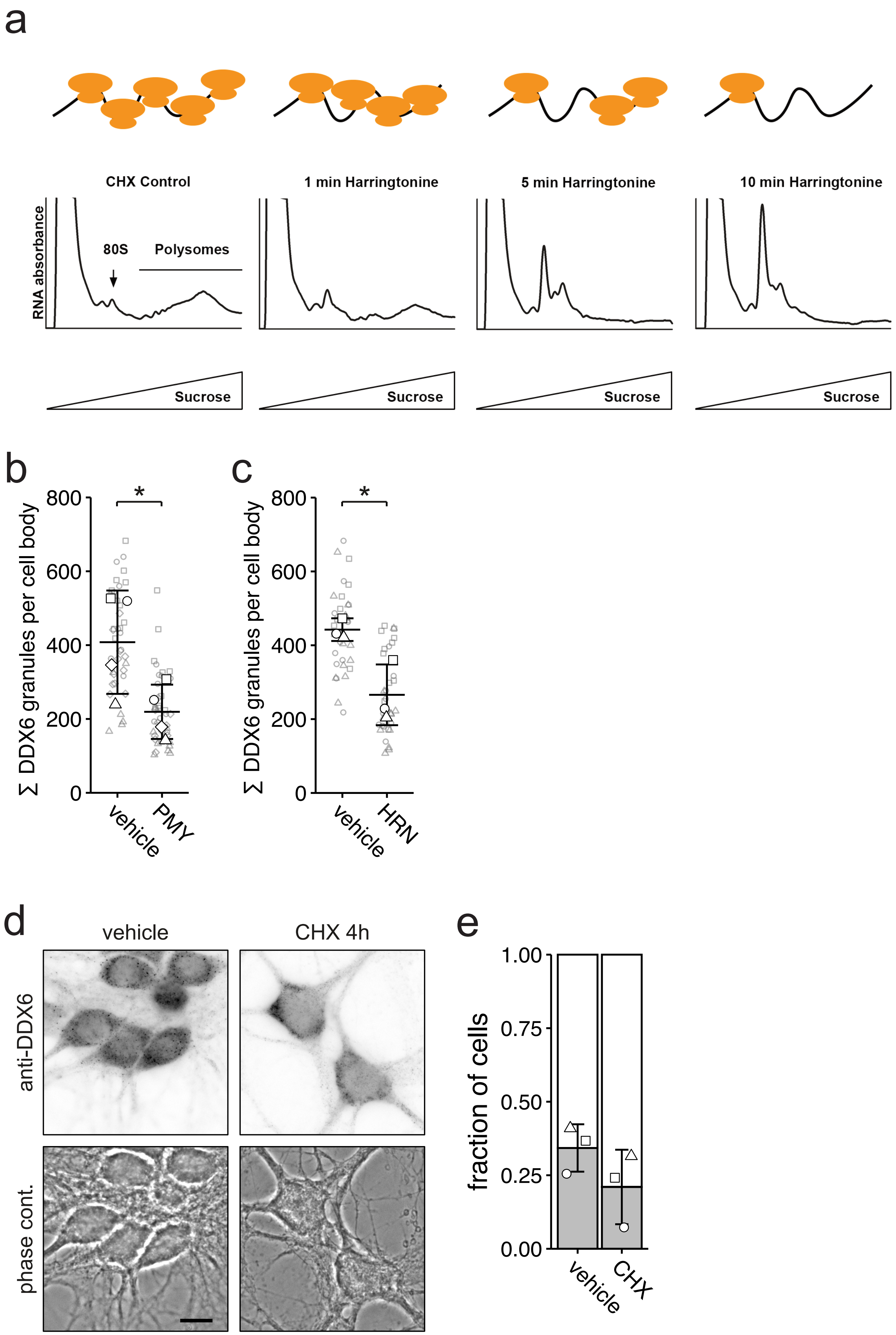

Supplementary Figure 4 - Bauer et al.

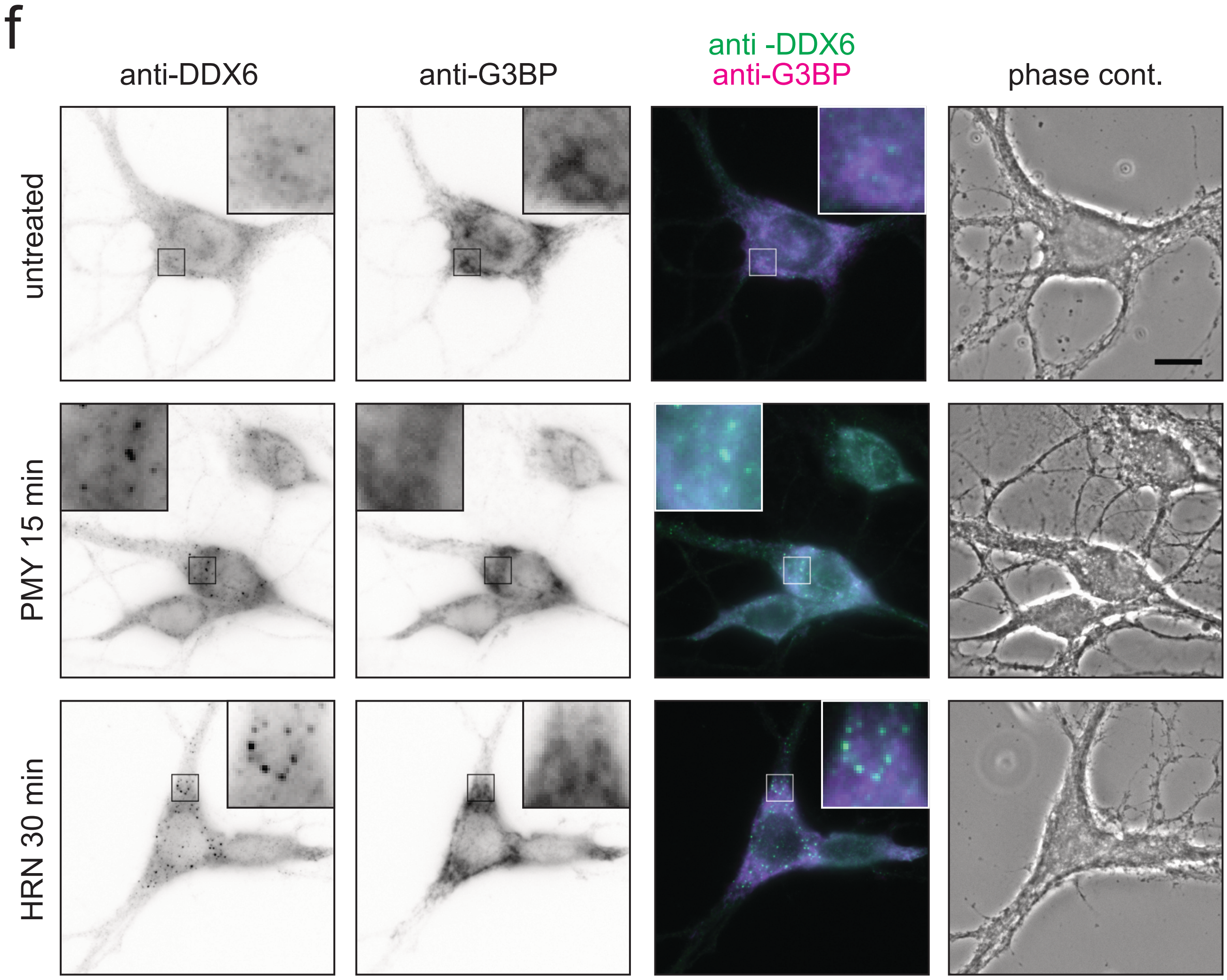
f
anti-DDX6
anti-G3BP
anti -DDX6
anti-G3BP
phase cont.
untreated
PMY 15 min
HRN 30 min

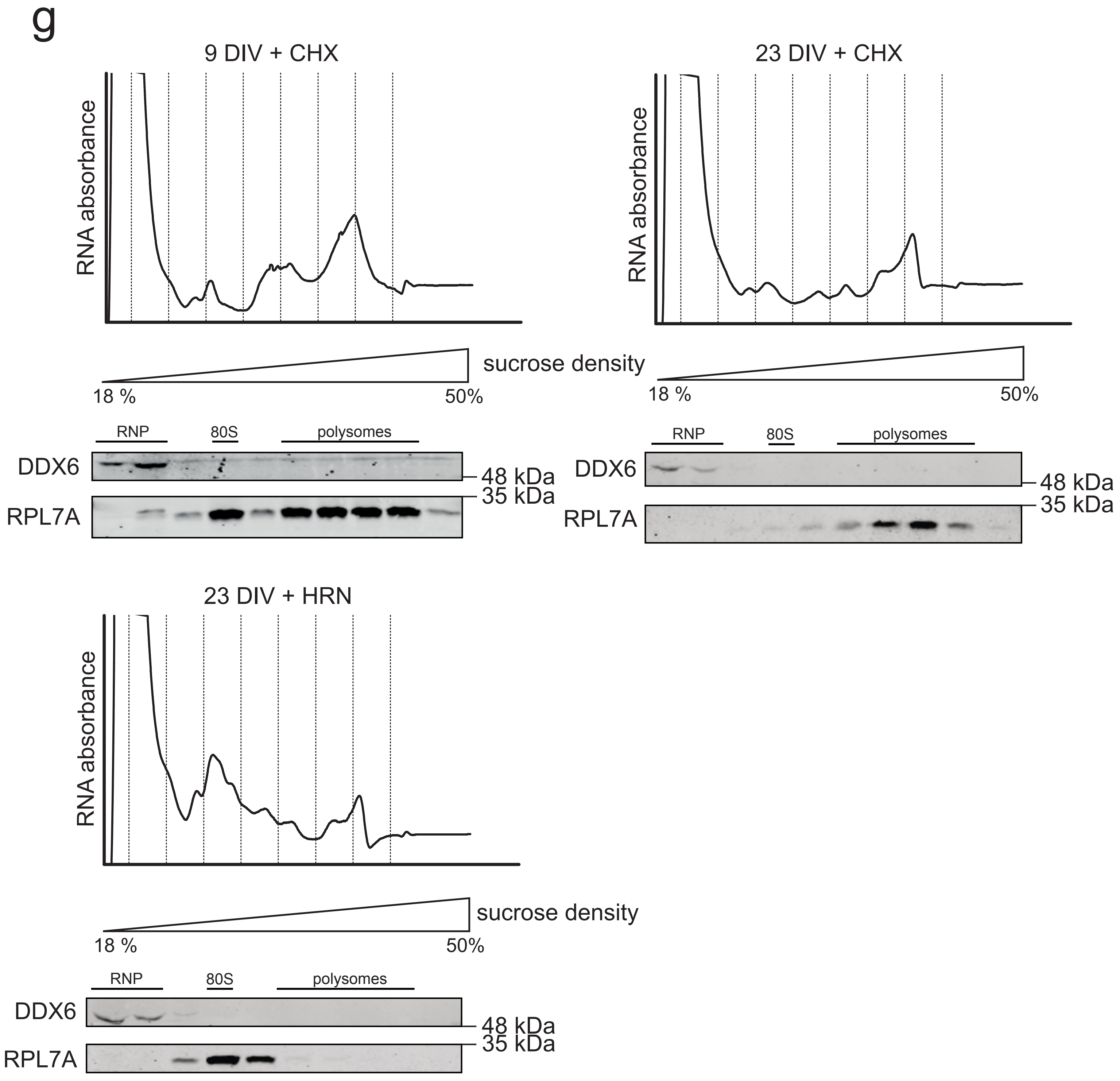

Supplementary Figure 4 (continued) - Bauer et al.

Supplementary Figure 4: DDX6 granules induced by puromycin or harringtonine are not stress granules and DDX6 is not found in polysome fractions associated with translating mRNA. (**a**) Representative polysome profiles of cortical neurons treated either 10 min with CHX or 1, 5 or 10 min with harringtonine. (**b, c**) Dot plots displaying DDX6 granule number of individual cell bodies of 22 DIV hippocampal neurons under vehicle, PMY or HRN treated conditions. Small grey symbols represent single cells while larger white symbols indicate the average of each replicate. Distinct dot symbols indicate biological replicates. Horizontal line and error bars represent mean of replicates and standard deviation (n=4 or n=3 biologically independent experiments). Asterisks represent p-values obtained by two-sided Student's t-test (* $p < 0.05$). $p = 0.0145$ (b), $p = 0.0273$ (c). (**d**) Representative example of DDX6 immunostaining and phase contrast pictures of a 22 DIV hippocampal neurons in culture after 4h CHX treated conditions. (**e**) Bar plot displaying quantification of cell population by fraction of cells containing either large or small DDX6 granules as exemplified in (d). At least 100 cells/condition/experiment were quantified for (e). Data represents mean ± standard deviation of three independent neuronal cultures in (b,c,e). (**f**) Representative examples of DDX6 and G3BP immunostainings and phase contrast of 22 DIV hippocampal neurons in culture under vehicle, 15 min PMY (25 µM) or 30 min HRN treated (2 µg/mL) conditions. Boxed regions in images are displayed as magnified insets. Scale bars 10 µm. (**g**). Representative polysome profiles and immunoblots for DDX6 and RPL7A of CHX or HRN treated cortical neurons at 9 and 23 DIV, respectively. RPL7A served as marker for ribosomes. Abbreviations: CHX=cycloheximide, PMY=puromycin, HRN=harringtonine, DIV=days *in vitro*, RPL7A=Ribosomal Protein L7A. *Related to Figure 4*.

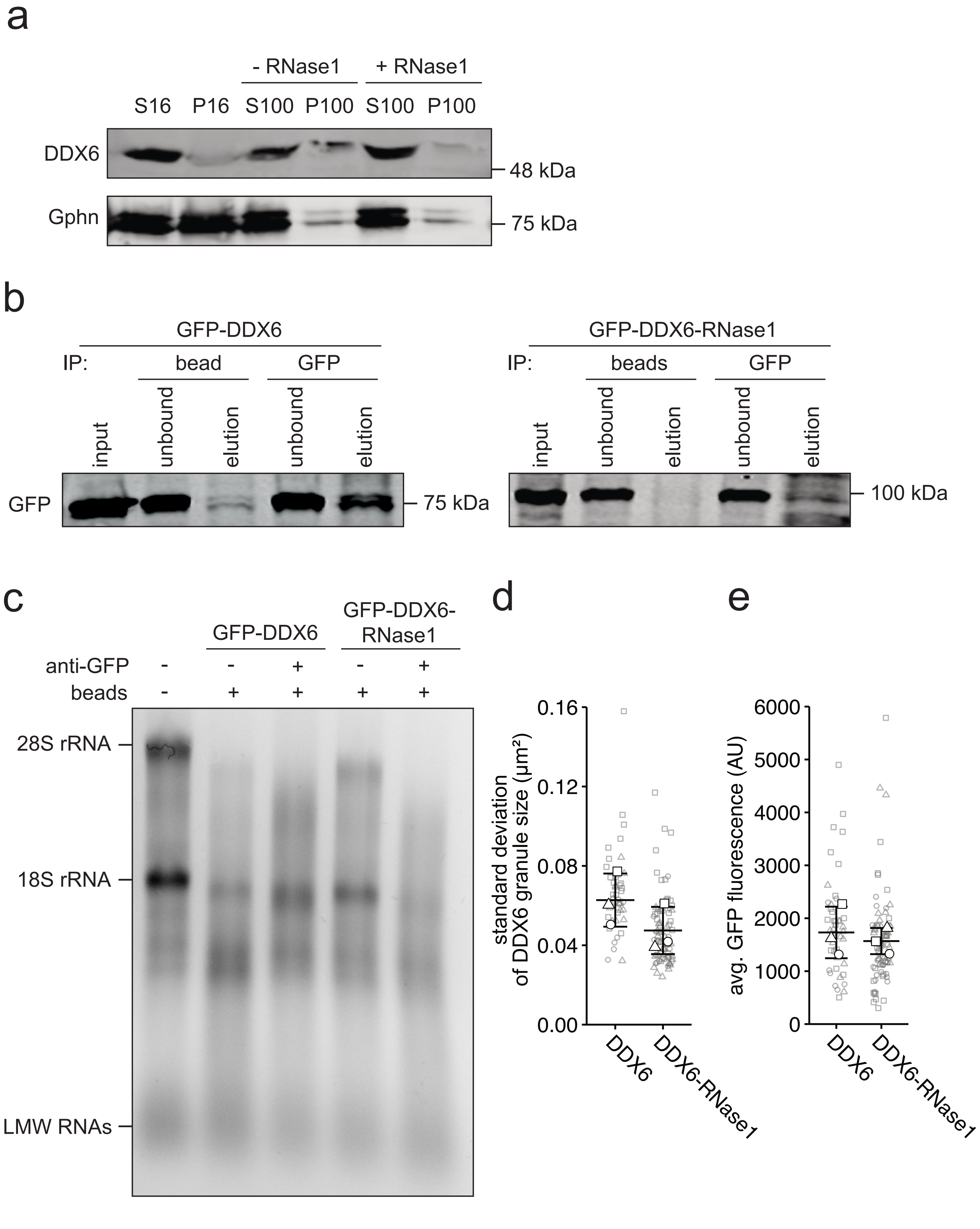

Supplementary Figure 5 - Bauer et al.

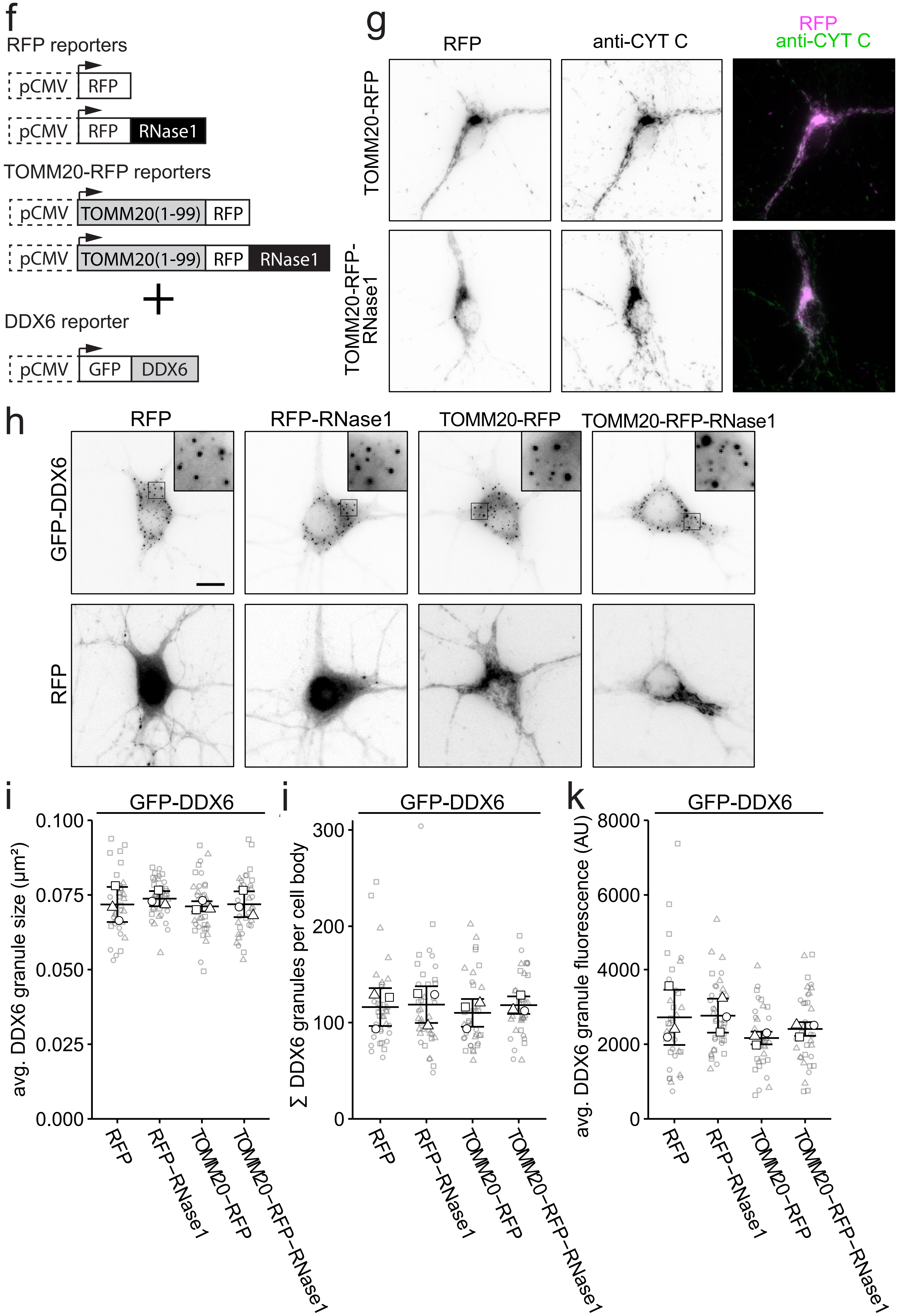

Supplementary Figure 5 (continued) - Bauer et al.

l

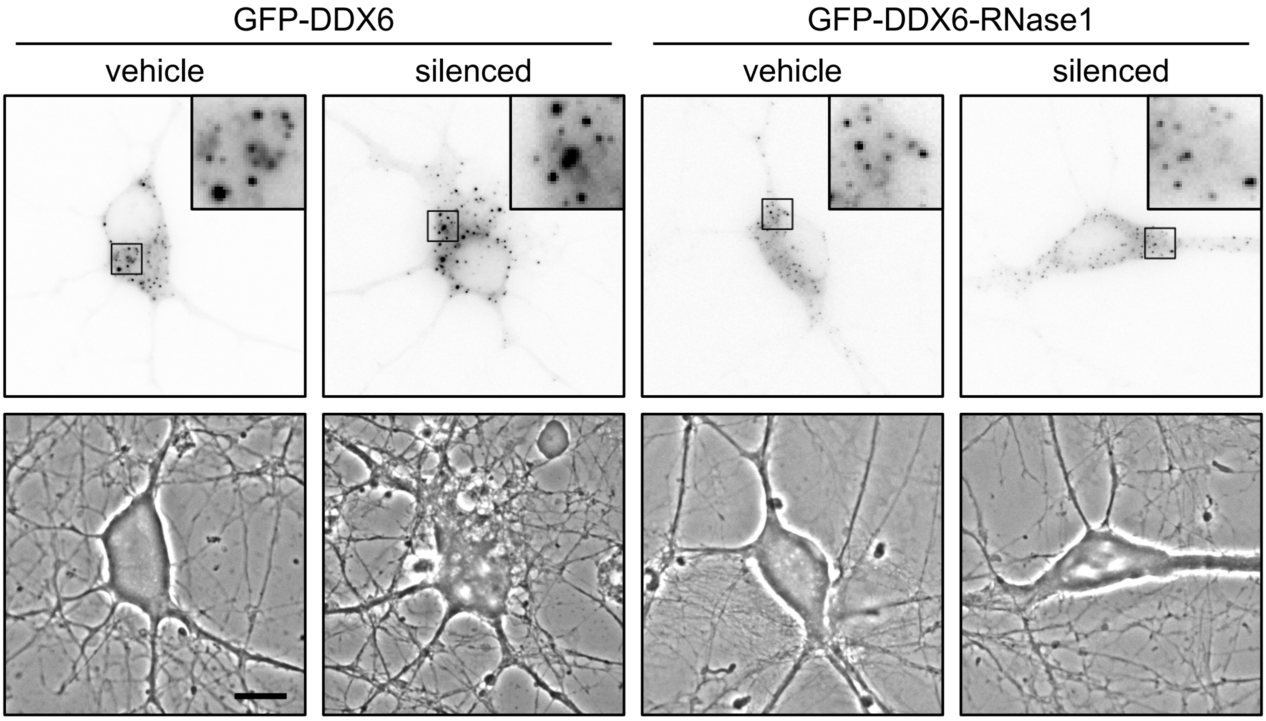

GFP-DDX6
GFP-DDX6-RNase1
vehicle
silenced
vehicle
silenced
GFP
phase cont.

m

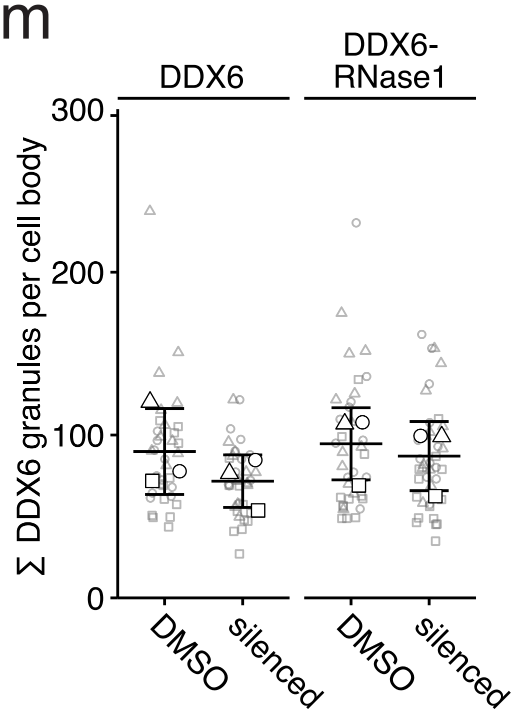

DDX6
DDX6-
RNase1
300
200
100
0
Σ DDX6 granules per cell body
DMSO
silenced
DMSO
silenced

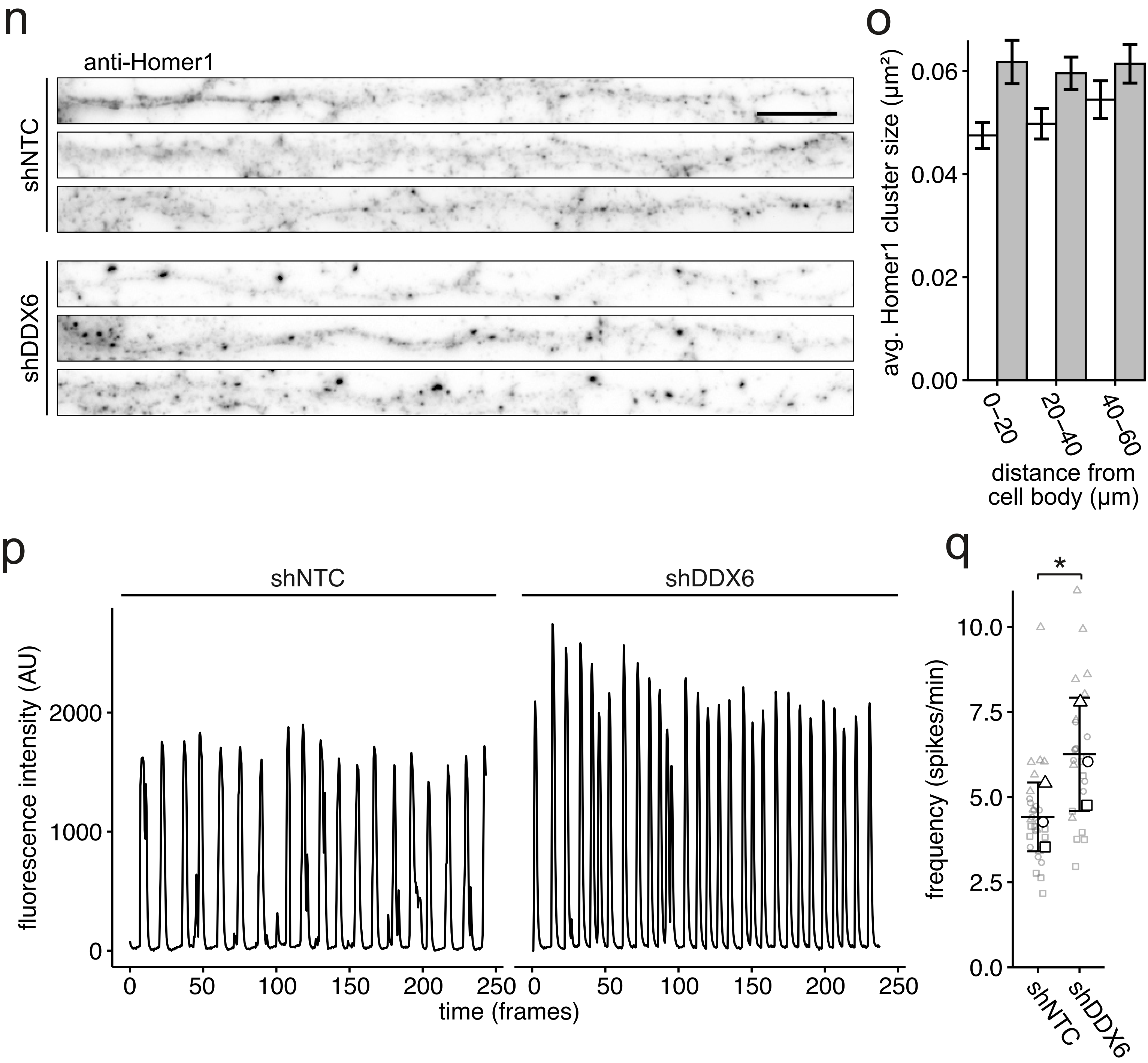

Supplementary Figure 5 (continued) - Bauer et al.

Supplementary Figure 5: RNA degradation results in DDX6 granule disassembly. (**a**) Representative Western blot for endogenous DDX6 and Gphn upon differential centrifugation assay with and without RNase1 treatment. Gphn=Gephyrin. (**b**) Western blot for GFP after control (beads) or anti-GFP immunoprecipitation (IP) of GFP-DDX6 (left) and GFP-DDX6-RNase1 (right) from lysates of transfected HEK 293T cells. (**c**) Formaldehyde agarose gel stained with ethidium bromide upon incubation of isolated total rat brain RNA with either GFP-DDX6 or GFP-DDX6-RNase1. Experiments were repeated 3 (a) or 2 (b,c) times independently with similar results. (**d, e**) Dot plot displaying standard deviation of DDX6 granule size (d) and mean GFP fluorescence (e) of individual cell bodies of hippocampal neurons transfected with either GFP-DDX6 or GFP-DDX6-RNase1 reporters (n=3 biologically independent experiments). AU = arbitrary units. (**f**) Scheme of RFP, RFP-RNase1, TOMM20-RFP, TOMM20-RFP-RNase1 and GFP-DDX6 constructs. (**g**) Representative examples of RFP fluorescence, anti-cytochrome C immunofluorescence and merged pictures of 17 DIV hippocampal neurons, transfected with either TOMM20-RFP or TOMM20-RFP-RNase1 reporters, respectively. (**h**) Representative examples of GFP and RFP fluorescence in 17 DIV hippocampal neurons, co-transfected with GFP-DDX6 and the indicated RFP reporters, respectively. (**i,j,k**) Dot plots displaying average DDX6 granule size (i), granule number per cell body (j) and average fluorescence intensity (k) of neurons as in (h) (n=3 biologically independent experiments). (**l**) Representative examples of GFP fluorescence and phase contrast pictures of hippocampal neurons in culture, transfected with either GFP-DDX6 or GFP-DDX6-RNase1 reporters under vehicle treated (DMSO) or silenced (100µM CNQX, 50µM AP5, 1µM TTX) conditions. Boxed regions in images are displayed as magnified insets. Scale bars 10 µm. (**m**) Dot plots displaying DDX6 granule number of individual cell bodies (n=3 biologically independent experiments). (**n**) Representative examples of anti-Homer1 immunofluorescence in dendrites of 14 DIV hippocampal neurons in culture transfected either with shNTC or shDDX6. Scale bar 10 µm. (**o**) Bar plot displaying average Homer1 cluster size in 20 µm segments binned along dendrites. Error bars = SEM. (**p**) Representative examples of Ca^{2+} sensor fluorescence intensity over time in 15 DIV hippocampal neurons transduced with shNTC or shDDX6. (**q**) Dot plot displaying average number of fluorescence intensity peaks per minute. Distinct dot symbols indicate individual biological replicates (n=3 biologically independent experiments). Small grey symbols represent single cells while larger white symbols

indicate the average of each replicate. Horizontal line and error bars represent mean of replicates and standard deviation. Asterisks represent p-values obtained by two-sided Student's t-test (* $p < 0.05$). $p = 0.043$ (c). *Related to Figure 5*.

Supplementary Movie 1: Representative time-lapse movie of an 11 DIV hippocampal neuron after overnight expression of GFP-DDX6. Boxed region magnified in right panel. Green arrowheads indicate fusing, red arrowheads indicate splitting events of GFP-DDX6 granules. Timestamp indicates hours:minutes. Scale bar 10 µm. *Related to Figure 1.*

Supplementary Movie 2: Representative time-lapse maximum intensity projection movie of a 16 DIV hippocampal neuron expressing GFP-DDX6 during incubation with 100µM NMDA or subsequent wash-off. Green arrowhead indicates disassembly of an individual granule, red arrowheads indicates assembly of an individual granule. Timestamp indicates minutes:seconds. Boxed regions indicate area of magnified insets displayed below. Scale bars 10 µm. *Related to Figure 2*.

Supplementary Movie 3: Representative time-lapse movie of shNTC or shDDX6 transduced 15 DIV hippocampal neurons after overnight expression of GCaMP6s for Ca^{2+} imaging. Timestamp indicates minutes:seconds. Scale bar 10 µm. *Related to Figure 5.*

uncropped blots and gels

Supplementary Fig. 1f

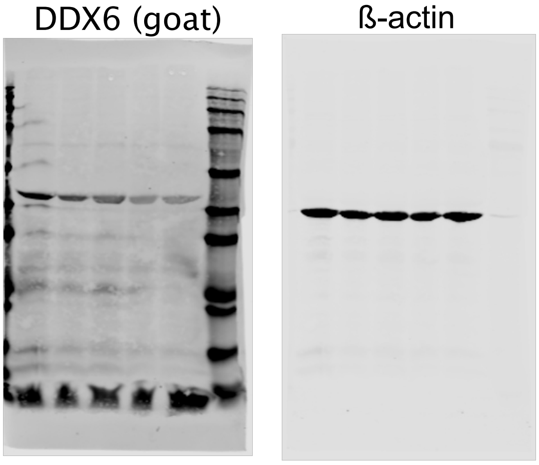

blot 1

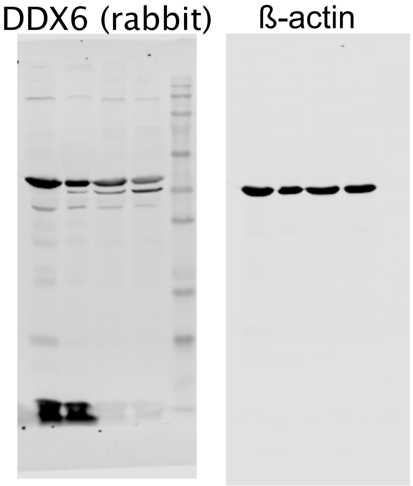

blot 2

Supplementary Fig. 4g

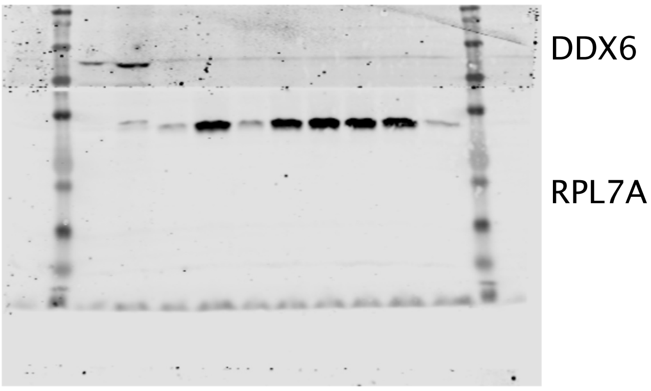

blot 1

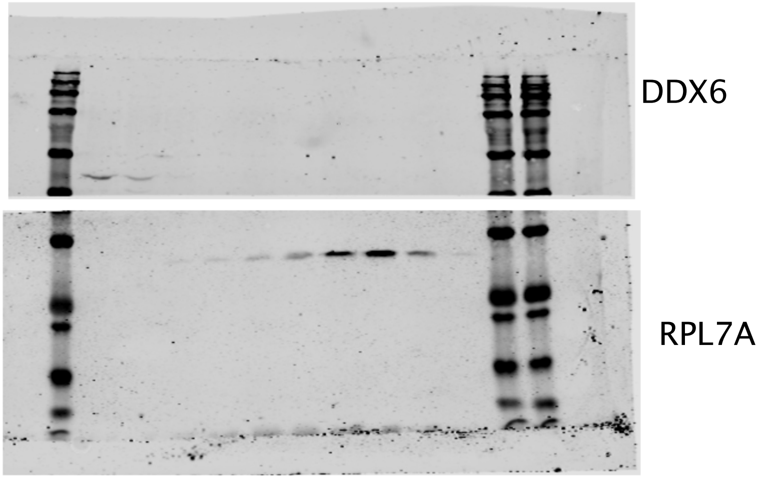

blot 2

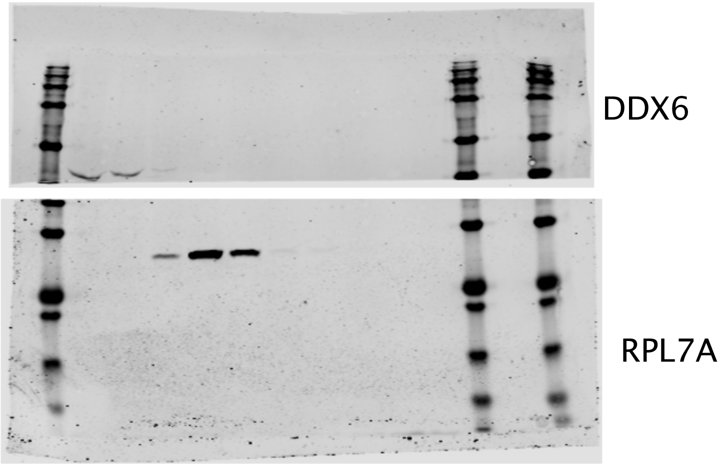

blot 3

uncropped blots and gels

Supplementary Fig. 5a

DDX6 Gphn

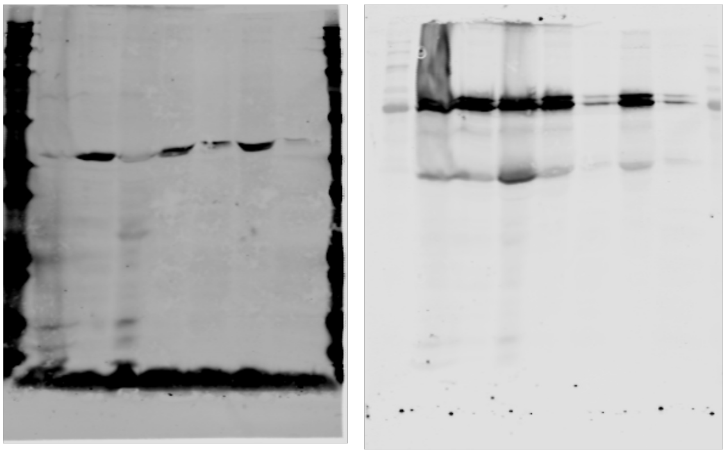

Supplementary Fig. 5b

GFP GFP

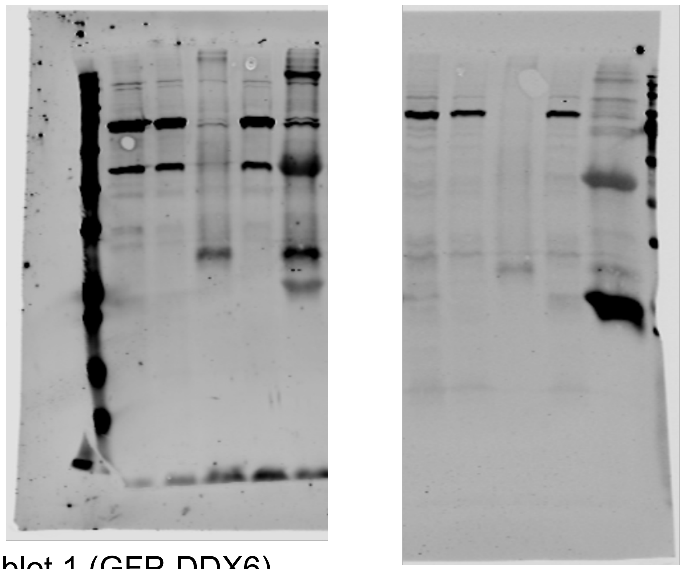

blot 1 (GFP-DDX6)

blot 2 (GFP-DDX6-RNase1)

Supplementary Fig. 5c

* RNA Millennium Markers (Applied Biosystems)

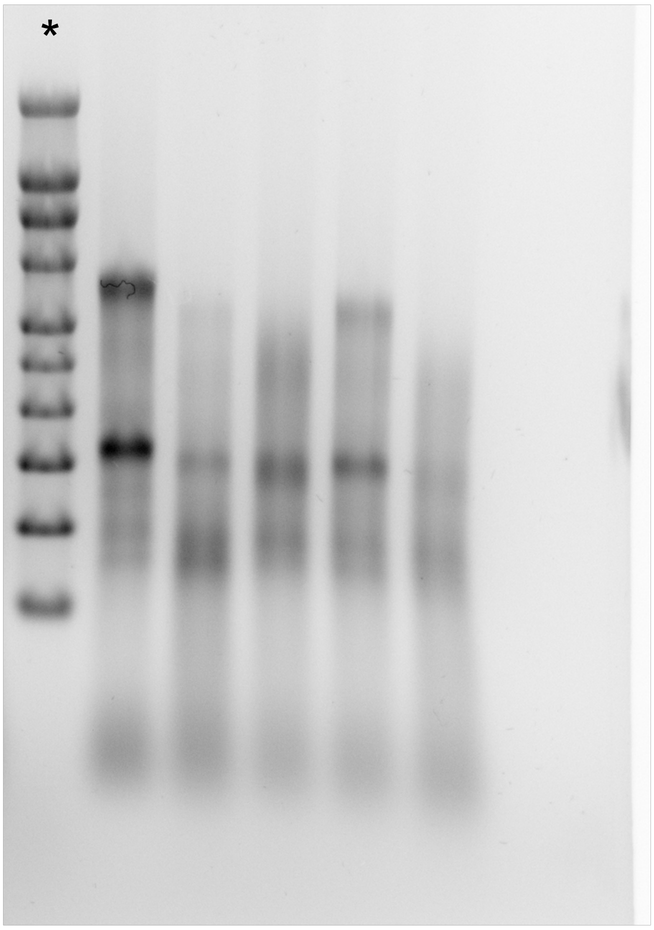

Supplement: Supplementary file 1 — Supplementary Information [file 41467_2022_30067_MOESM1_ESM.pdf]
