# Supplementary material for: RNA supply drives physiological granule assembly in neurons
Source: Nat Commun. 2022 May 19;13:2781. doi: 10.1038/s41467-022-30067-3 (PMC9120520; doi:10.1038/s41467-022-30067-3)
Supplement: Supplementary file 3 — Description of Additional Supplementary Files [file 41467_2022_30067_MOESM3_ESM.pdf]

Description of Additional Supplementary Files

File Name: Supplementary Movie 1
Description: Representative time-lapse movie of an 11 DIV hippocampal neuron after overnight expression of GFP-DDX6. Boxed region magnified in right panel. Green arrowheads indicate fusing, red arrowheads indicate splitting events of GFP-DDX6 granules. Timestamp indicates hours:minutes. Scale bar 10 µm. *Related to Figure 1.*

File Name: Supplementary Movie 2
Description: Representative time-lapse maximum intensity projection movie of a 16 DIV hippocampal neuron expressing GFP-DDX6 during incubation with 100µM NMDA or subsequent wash-off. Green arrowhead indicates disassembly of an individual granule, red arrowheads indicates assembly of an individual granule. Timestamp indicates minutes:seconds. Boxed regions indicate area of magnified insets displayed below. Scale bars 10 µm. *Related to Figure 2*.

File Name: Supplementary Movie 3
Description: Representative time-lapse movie of shNTC or shDDX6 transduced 15 DIV hippocampal neurons after overnight expression of GCaMP6s for Ca^{2+} imaging. Timestamp indicates minutes:seconds. Scale bar 10 µm. *Related to Figure 5.*
