# Supplementary material for: RNA supply drives physiological granule assembly in neurons
Source: Nat Commun. 2022 May 19;13:2781. doi: 10.1038/s41467-022-30067-3 (PMC9120520; doi:10.1038/s41467-022-30067-3)
Supplement: Supplementary file 7 — Reporting Summary [file 41467_2022_30067_MOESM7_ESM.pdf]

Corresponding author(s): Michael A Kiebler
Last updated by author(s): Mar 22, 2022

Reporting Summary

Nature Research wishes to improve the reproducibility of the work that we publish. This form provides structure for consistency and transparency in reporting. For further information on Nature Research policies, see our Editorial Policies and the Editorial Policy Checklist.

Statistics

For all statistical analyses, confirm that the following items are present in the figure legend, table legend, main text, or Methods section.

n/a	Confirmed	
☐	☒	The exact sample size (*n*) for each experimental group/condition, given as a discrete number and unit of measurement
☐	☒	A statement on whether measurements were taken from distinct samples or whether the same sample was measured repeatedly
☐	☒	The statistical test(s) used AND whether they are one- or two-sided<br>*Only common tests should be described solely by name; describe more complex techniques in the Methods section.*
☒	☐	A description of all covariates tested
☐	☒	A description of any assumptions or corrections, such as tests of normality and adjustment for multiple comparisons
☐	☒	A full description of the statistical parameters including central tendency (e.g. means) or other basic estimates (e.g. regression coefficient) AND variation (e.g. standard deviation) or associated estimates of uncertainty (e.g. confidence intervals)
☐	☒	For null hypothesis testing, the test statistic (e.g. *F*, *t*, *r*) with confidence intervals, effect sizes, degrees of freedom and *P* value noted<br>*Give P values as exact values whenever suitable.*
☒	☐	For Bayesian analysis, information on the choice of priors and Markov chain Monte Carlo settings
☒	☐	For hierarchical and complex designs, identification of the appropriate level for tests and full reporting of outcomes
☒	☐	Estimates of effect sizes (e.g. Cohen's *d*, Pearson's *r*), indicating how they were calculated

Our web collection on statistics for biologists contains articles on many of the points above.

Software and code

Policy information about availability of computer code

Data collection	ZEN 2.0, ZEN 3.0 (Zeiss), Arivis Vision 4D, ImageJ (Fiji) 2.0.0-rc-69/1.52p, ImageStudioLite 4.0.21
Data analysis	R 4.1.0, RStudio 1.4.1717, GraphPad Prism 5

For manuscripts utilizing custom algorithms or software that are central to the research but not yet described in published literature, software must be made available to editors and reviewers. We strongly encourage code deposition in a community repository (e.g. GitHub). See the Nature Research guidelines for submitting code & software for further information.

Data

Policy information about availability of data

All manuscripts must include a data availability statement. This statement should provide the following information, where applicable:
- Accession codes, unique identifiers, or web links for publicly available datasets
- A list of figures that have associated raw data
- A description of any restrictions on data availability

Source data are provided with this paper. The data generated in this study have been deposited at: https://osf.io/c7s6p/

Field-specific reporting

Please select the one below that is the best fit for your research. If you are not sure, read the appropriate sections before making your selection.

☒ Life sciences ☐ Behavioural & social sciences ☐ Ecological, evolutionary & environmental sciences

For a reference copy of the document with all sections, see nature.com/documents/nr-reporting-summary-flat.pdf

Life sciences study design

All studies must disclose on these points even when the disclosure is negative.

Sample size	For experiments involving manual quantification at the microscope, at least 100 cells/condition/experiment were analyzed. For experiments involving image analysis at least 10 cells/condition/experiment were quantified. 3-4 experiments were conducted, according to standard scientific practice for all experiments. Sample size was chosen according to Lord et al., JCB, 2020, PMID: 32346721.
Data exclusions	No data was excluded from analysis, unless cell health/viability was not adequate.
Replication	3-4 biological replicates (i.e. independent preparations of rat neurons) were performed. In cases where cell health was not adequate, cultures were not used for experiments. All attempts at replication were successful.
Randomization	All analysis was performed blind or automated.
Blinding	Microscopy images were acquired blindly. All experiments were analyzed blindly or automated for all conditions.

Reporting for specific materials, systems and methods

We require information from authors about some types of materials, experimental systems and methods used in many studies. Here, indicate whether each material, system or method listed is relevant to your study. If you are not sure if a list item applies to your research, read the appropriate section before selecting a response.

Materials & experimental systems

n/a	Involved in the study	
☐	☒	Antibodies
☐	☒	Eukaryotic cell lines
☒	☐	Palaeontology and archaeology
☐	☒	Animals and other organisms
☒	☐	Human research participants
☒	☐	Clinical data
☒	☐	Dual use research of concern

Methods

n/a	Involved in the study	
☒	☐	ChIP-seq
☒	☐	Flow cytometry
☒	☐	MRI-based neuroimaging

Antibodies

Antibodies used	Polyclonal antibodies: rabbit anti-Rck (MBL,# PD009) 1:500 IF/1:1000 WB, goat anti-DDX6 (Abnova, #PAB6971) 1:500 IF/1:1000, WB mouse anti-DCP1a (Abnova, #H00055802-A01-50) 1:500, rabbit anti-G3BP1 (Proteintech,#13057-2-AP) 1:500, rabbit anti-RPL7A (Abcam,# ab155147) 1:1000.<br>Monoclonal Antibodies: mouse anti-Stau2 1:500 (self made, clone7C3), mouse anti-Gephyrin (Synaptic Systems,#147-011, clone mAb7a) 1:1000, mouse anti-ß-actin (Sigma-Aldrich,#A2228, clone AC-74) 1:5000, monoclonal mouse anti-CYT C 1:200 (Biolegend,#612302, clone: 6H2.B4), mouse anti-GFP (self-made, kind gift by Angelika Noegel, Köln, clone K64-372-2) 1:500, mouse monoclonal anti-Homer1 1:500 (Synaptic Systems,#160-011, clone 2G8)<br>Secondary antibodies: donkey anti-rabbit or donkey anti-mouse AlexaFluor488(#A21206,#A20202) 1:1000, AlexaFluor555 (#A31570,# A31572)1:1000 or AlexaFluor647 (#A31573, #A31571) 1:1000, conjugated antibodies (Life Technologies), donkey anti-rabbit IRDye 680RD conjugated (LI-COR,#926-68073) 1:10000, donkey anti-mouse IRDye 800CW conjugated (LI-COR,#926-32212) 1:10000, donkey anti-goat IRDye 680RD conjugated (LI-COR,#926-68074) 1:10000.
Validation	Fritzsche, R. et al. Interactome of two diverse RNA granules links mRNA localization to translational repression in neurons. Cell Reports 5, 1749-1762 (2013). for mouse anti-Stau2 (self made) and goat anti-DDX6 (Abnova)<br>This study (Supplementary Figure 3). for mouse anti-Stau2 (self made)

Eukaryotic cell lines

Policy information about cell lines

Cell line source(s)	primary rat hippocampal and cortical neurons, HEK-293T ATCC CRL-3216

Authentication	for primary rat hippocampal neurons in house isolation including validation, HEK-293T (ATCC CRL-3216, their GMP)
Mycoplasma contamination	Cell cultures are checked for mycoplasma contamination at regular intervals.
Commonly misidentified lines (See ICLAC register)	No commonly misidentified lines were used in this study.

Animals and other organisms

Policy information about studies involving animals; ARRIVE guidelines recommended for reporting animal research

Laboratory animals	Embryonic day 17 (E17) embryos of timed pregnant Sprague-Dawley rats (Charles River Laboratories), male Black 6 mice 8-day or 10-month old (C57B/6J, Jackson Laboratory). Mice were kept under specified pathogen-free conditions and housed in groups of 2–5 animals with a 12-h light/12-h dark light cycle in individually ventilated cages, at 22 +/- 2 °C and 55 +/- 10 % relative humidity. Animals had free access to autoclaved water and food.
Wild animals	No wild animals were used in this study.
Field-collected samples	No field collection samples were used in this study.
Ethics oversight	All animals were used according to the German Welfare for Experimental Animals (LMU-Munich, Regierung von Oberbayern).

Note that full information on the approval of the study protocol must also be provided in the manuscript.
